# Literature Review on the Effects of Heavy Metal Stress and Alleviating Possibilities through Exogenously Applied Agents in Alfalfa (*Medicago sativa* L.)

**DOI:** 10.3390/plants11162161

**Published:** 2022-08-20

**Authors:** Ildikó Jócsák, Bence Knolmajer, Miklós Szarvas, Gyula Rabnecz, Ferenc Pál-Fám

**Affiliations:** 1 Institute of Agronomy, Kaposvár Campus, Hungarian University of Agriculture and Life Sciences, Guba Sándor Street 40, H-7400 Kaposvár, Hungary; 2 Institute of Plant Protection, Georgikon Campus, Hungarian University of Agriculture and Life Sciences, Deák Ferenc Street 16, H-8360 Keszthely, Hungary; 3 Zorvet Ltd., Wlassics Gyula Street 58, H-1181 Budapest, Hungary

**Keywords:** alfalfa, heavy metal stress, stress alleviation, exogenously applied agents

## Abstract

Heavy metals (HMs) are among the most important toxic agents since they reach the soil through various routes and accumulate in the food chain. Therefore, HMs induce problems in soil integrity and in plant, animal, and human health. Alfalfa (*Medicago sativa* L.) is a significant crop worldwide, utilized in animal production. Furthermore, because of its nitrogen-absorbing ability via symbiotic strains of bacteria, it increases soil productivity. However, there are relatively few studies investigating the effects of HMs and their alleviation possibilities on alfalfa plants. Therefore, the goal of this review is to clarify the current state of research into HM-induced alterations in alfalfa and to determine the extent to which externally applied microorganisms and chemical compounds can mitigate the negative effects. The aim is to indicate areas of development towards further understanding of HM detoxification in alfalfa and to identify future research directions.

## 1. Introduction

Human industrial and agricultural activity leads to increased levels of environmental contaminants that affect the productivity and quality of crop plants worldwide. Therefore, it is vital to understand and study contaminants’ harmful effects and the possibilities of neutralization and elimination [1]. Toxic substances released into the biosphere affect all living organisms, including plants. The release of heavy metals (HMs) poses one of the most significant problems for soil integrity and plant health. The entry of possibly contaminated crops into feed or food markets poses global risks [1,2,3,4,5]. Alfalfa (*Medicago sativa* L.) is an important forage legume cultivated on about 32 million hectares worldwide [6]. It is also important for sustainable farming since it increases soil fertility due to the nitrogen-absorbing capability associated with the symbiotic traits of *Rhizobium* bacteria [7,8,9,10]. As a consequence of this symbiosis, alfalfa is able to fix 60–80 kg of atmospheric N per hectare per year [11]. Moreover, its roots are deeply penetrating, thus supplying calcium, potassium, and phosphorus from the deeper soil levels, enriching the fertile topsoil area [12]. Additionally, it protects the soil against water and deflation by providing constant cover throughout the year and for several years; furthermore, alfalfa is the highest-yielding leguminous fodder crop, containing high levels of protein per unit area [12]. Therefore, its industrial usage is significant because of its beneficial properties and high nutritional value. Its main role is in hay production, but it can also be used to make silage or as a pasture crop. Studies are also being conducted to find more ways to use the plant, such as to generate electricity [13].

As a member of the legume plant family, alfalfa and its associated Gram-negative soil bacteria (*Rhizobium* spp.) are severely impacted by HMs because the HMs reduce the efficiency of symbiosis and, consequently, alfalfa’s ability to fix nitrogen [10,14].

Recent efforts to protect plants and the environment have led to the reduction in the negative effects of pollutants on soil and plants. This has given rise to the relatively new practice of stress mitigation, which involves the external administration of plant compounds or organisms to improve plant homeostasis or increase the resistance of plants to environmental stressors.

In contrast to major crop plants such as wheat and maize, alfalfa plants have been the subject of relatively few studies examining the effects of HMs and the potential for their mitigation. Therefore, the purpose of this review was to clarify the current state of knowledge regarding HM effects on alfalfa and to elucidate the applicability of exogenously administered stress-alleviating microorganisms and chemical substances in order to provide an in-depth contribution towards further understanding of HM detoxification.

## 2. Definition of Heavy Metals and Their Sources

Plant production is affected by a number of detrimental environmental factors [15], one of the most important of which is HM pollution. The term “heavy metal” has generated a large amount of controversy in the scientific community [16,17,18] and has different definitions and interpretations in various fields of science, such as chemistry or plant sciences [18]. Based on these definitions, the group of chemical elements with metallic properties is generally referred to as the HMs [16]. The present work does not aim to revisit or resolve the issue of the group’s definition, which has already generated widespread scientific debate. However, the categorization based on density (3.5–7 g/cm^3^) or atomic number (greater than 20) appears to be have been replaced by a definition related to the mode of action in plant sciences [18]. In their work, Pourret and Bollinger (2018) propose the terms “potentially toxic metal(s)/element(s)” or “trace metal(s)/element(s)” [17]. They do, however, point out that the term “potentially toxic metal(s)/element(s)” is not precise enough. However, from a plant physiological aspect, this definition might adequately delineate the problem that these elements pose to plants.

**Figure 1:**
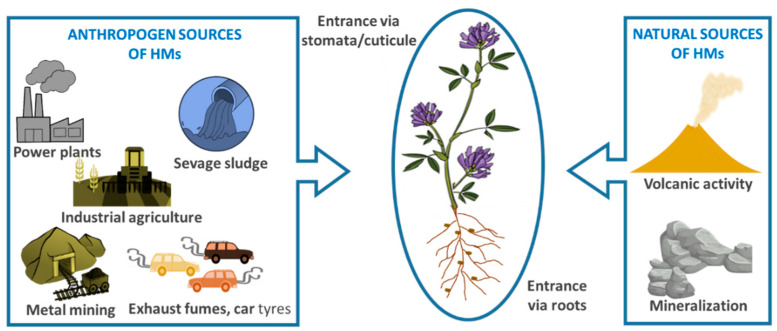
Anthropogenic and natural sources of heavy metals in the environment.

Soil contaminated with wastewater in both urban and agricultural areas can be contaminated with HMs released from increased industrial activity, and the resulting sewage sludge infiltrating into the soil can pose very serious risks. Aerosol particles from the combustion of fossil fuels or other sources are another source of HM pollution that can easily contaminate our agricultural areas by spreading in the atmosphere. Moreover, the increased use of phosphate fertilizers [19] also affects the contamination levels of soils and, thus, long-term usage of these fertilizers involves high levels of HMs. Recently, strict regulations have led to the decreased release of toxic HMs into the soil. Regulatory examples include decreased atmospheric emissions, limitations on the HM content of wastewater, and lead removal from paints and fuels [1] (Figure 1).

The Regulation of the European Parliament and of the European Council set the rules for the marketing of fertilizers in the EU, and came into force on 16 July 2022. This regulation severely limits the concentration of HMs in fertilizers that can be used, including cadmium (Cd), nickel (Ni), chromium (Cr), mercury (Hg), lead (Pb), and arsenic (As) ((EU)2019/1009, 2019). It is expected that the above-mentioned regulation will reduce the concentration of HMs in agricultural soils. From the above, it is seen that efforts to reduce the presence of cadmium and other contaminating HMs in plants are of utmost importance.

HMs have two main categories: essential and non-essential HMs. The most important essential HMs functioning as microelements are zinc (Zn), cobalt (Co), manganese (Mn), molybdenum (Mo), nickel (Ni), copper (Cu), vanadium (V), and iron (Fe). They play a dominant role in living organisms but may be toxic at high concentrations [15]. Microelements are necessary only in minute amounts for plant metabolism, but they are of great physiological importance: they can be active sites for enzymes or atoms of prosthetic groups [20]. There are two potential concerns with essential elements: a deficiency that may lead to a failure of plants to develop, usually resulting in a reduction in photosynthetic production and, ultimately, a lower yield. In the case of excess, which may be due to high doses and/or prolonged low-dosage poisoning, toxic symptoms occur, eventually leading to lethality [21].

**Figure 2:**
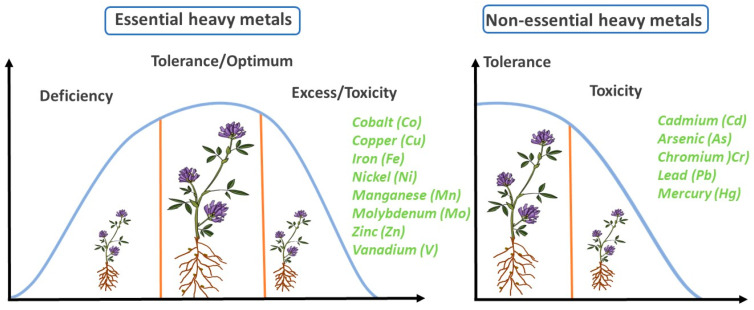
Mode of action of essential and non-essential heavy metals.

In terms of their effects, one of the most common consequences of stress caused by HMs is the inhibition of plant growth [12] (Figure 2) as result of several metabolic processes, as described later. Tolerance to essential elements can typically be described by a Gaussian curve, in the initial phase of which the plant metabolism is in a stimulatory phase, which is the category and period of eustress according to the stress concept of Hans Selye proposed in 1975 [22]. When the presence of essential HMs persists in the plant’s environment or the concentration is sufficiently high, the plant’s ability to adapt is exceeded by the stressor. This is followed by the decline in both metabolic processes and productivity leading to plant exhaustion or, in the worst case, death of the plant. For non-essential heavy metals, only a decrease in production is observed after a plateau-like tolerance phase [23] (Figure 2).

## 3. Effects of Essential Heavy Metals on the Growth and Development of Alfalfa

***Zinc******(Zn):*** excess level of Zn results in growth inhibition, leaf chlorosis, and depletion of photosynthetic rates as a result of metal toxicity. Bandyopadhyay et al. (2015) [2] found that a high amount of Zn (750 mg/kg soil) accumulates mostly in roots (300–400 mg kg^−1^ DW), but a significant amount also reaches stems (30–60 mg kg^−1^ DW) and leaves (20–45 mg kg^−1^ DW). In the work of Ibekwe et al. (1996) [24], 10 days of exposure of 4–7.3 mM Zn treatment resulted in chlorotic symptoms with necrotic spots and poor root development. Yahagi et al. (2019) found that 1.5–24 mM Zn significantly reduced germination rates [25].

***Manganese (Mn):*** Li et al. (2019) [26] summarized the symptoms of Mn toxicity as browning of roots and disruption of other nutrient uptake; interveinal chlorosis in young leaves; necrotic spots in mature leaves; and generally triggered oxidative stress symptoms. Sale et al. (1992) [27] found that severe Mn toxicity (60 mg L^−1^) resulted in a reduction in dry weight by 20% compared to that of the control. Gherardi and Rengel (2003) found that 500 µg g^−1^ Mn significantly decreased the growth of shoots and roots of all the tested genotypes, and the results showed a strong correlation of the alfalfa root: shoot dry weight at low, adequate, and high Mn applications (R^2^ = 0.97) [28].

***Molybdenum (Mo):*** Although there are not many references regarding Mo toxicity on alfalfa, Jensen et al. (1970) reported that an excess amount of Mo on forage plants generally decreased dry weight, but there was a significant difference between the Mo accumulating capability of forage plants, such as birdsfoot trefoil and alfalfa [29].

***Nickel (Ni):*** The role of nickel in plant metabolism has long been controversial, but it has now been shown to be an essential HM, found in glyoxalase enzymes, peptide deformylases, the porphyrin compound F430, the central metal atom of methyl-Co M reductase, and some superoxide dismutases and hydrogenases. Its essential nature was first demonstrated in legumes as a constituent of the enzyme urease [30], and nickel is also involved in the activation of this enzyme [31]. Subsequently, urease has been detected in all plant tissues [32], its primary function being to enable the organism to use urea to produce nitrogen. The enzyme urease (EC 3.5.1.5, urea amidohydrolase) is found in the cytosol [33], a nickel-dependent metalloenzyme that catalyzes the hydrolysis of urea to ammonia and carbon dioxide [34]. Alfalfa is able to absorb a considerable amount of nickel. Sixty days of Ni exposure (0, 50, 150, 250, and 500 mg kg^−1^) significantly increased peroxidase (POX) and glutathione-S-transferase (GST) activities and MDA levels. Moreover, Prx1C, GST, and phytochelatin synthase (PCs) genes were also up-regulated in shoots and roots as well [35].

***Copper (Cu):*** Copper poses a constraint on the reproduction and growth of diazotroph bacteria responsible for nitrogen fixation in alfalfa [36]. The plants’ stem apoplasts accumulate copper under high availability, probably influencing the cell wall properties of the stem that affect the quality of alfalfa. Thereafter, it leads to a reduced iron concentration in the stem and a reduced level of ferritins, which are a ubiquitous group of proteins responsible for the control of Fe levels in the redox status of the cells [37]; the role of ferritins in Cu homeostasis is yet to be identified [10,38].

***Vanadium (V):*** V is the 22nd most-abundant element, and the fifth most-abundant transitional metal [39]. The toxicity of V is recognized to be comparable to that of mercury, lead, and arsenic [5]. It is mainly released into the soil during the process of mineral resource misuse, which may lead to mass accumulation. One example of this is a vanadium-bearing titanomagnetite reserve in China (Panzhihua), where the V content in surface soils increased to 4793.6 mg kg^−1^, compared to the normal soil background value of V in China (82 mg kg^−1^) [40].

**Figure 3:**
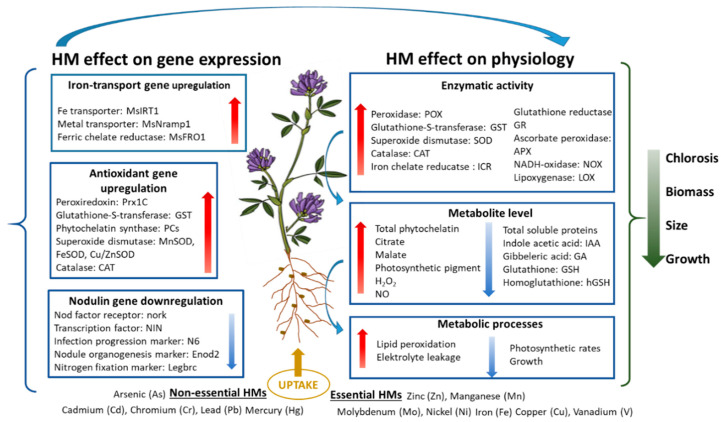
Effects of essential and non-essential heavy metals on alfalfa.

***Iron:*** Although a large amount of research has been conducted on iron toxicity in rice, the review of the research showed that no research has been undertaken on iron toxicity in alfalfa [41] (Figure 3, Table 1).

## 4. Effects of Non-Essential Heavy Metals on the Growth and Development of Alfalfa

The effects of non-essential elements have only a negative impact on plant metabolism and, after a narrow tolerance phase, the plant is finally in a phase of exhaustion. It can therefore be said that, even at low concentrations, the presence of non-essential HMs in the plant is a burden and triggers destructive processes. Non-essential HMs such as arsenic (As), cadmium (Cd), chromium (Cr), lead (Pb), and mercury (Hg) can be incorporated into some organisms and displace essential metals, thus causing damage to the organism [42].

***Arsenic (As):*** As affects the expression of nodulation genes that have been associated with processes that take place in the epidermis and the outer cortical cells [42]. A quantity of 25–35 M arsenate led to a decreased infective root zone, resulting in a 75% decrease in nodules due to the reduction in rhizobial infections, but the development of the established nodules seemed to grow normally, although they showed early senescence symptoms [43].

***Cadmium (Cd):*** Cd toxicity also reduces nutrients and water uptake in some crops. Cd concentrations of 3 and 5 mg/kg soil significantly reduced the presence of K, Mg, Ca, and Fe, in addition to root and shoot length and dry matter accumulation characteristics [44]. It retards germination processes. It also inhibits seedling growth after germination [24]. It also inhibits the morphological and physiological development and function of plants, and disrupts plant metabolism [44,45]. Cd has also been shown to cause other negative effects including oxidative stress, genotoxicity, inhibition of photosynthetic function, and inhibition of root metabolism [46]. Kabir et al. (2016) investigated Cd stress-related gene expression, enzyme activity, and metabolite levels in alfalfa and revealed that 1 mM Cd lowered the level of total soluble proteins and enhanced electrolyte leakage, iron chelate reductase activity, and total phytochelatin, citrate, and malate levels in roots and shoots of 7-day-old alfalfa seedlings along with the up-regulation of three Fe-related genes: Fe transporter (MsIRT1), metal transporter (MsNramp1), and ferric chelate reductase (MsFRO1). Due to Cd stress, the enzyme activity of catalase (CAT), ascorbate peroxidase (APX), and superoxide dismutase (SOD) in the roots of tolerant alfalfa decreased significantly in tolerant lines, while the content of H_2_O_2_ increased as a sign of oxidative stress [47]. Similarly, in the work of García de la Torre (2021), the presence of Cd increased dry weight and percentage of relative germination in tolerant lines of *Medicago truncatula*, whereas a reduced amount of hydrolyzed starch and resulted in unchanged SOD activity as a sign of increased tolerance level in shoots along with a higher level of H_2_O_2_ [48]. However, a lower level of lipid peroxidation and the loss of plasma membrane integrity was found in roots, in addition to the above-mentioned parameters for shoots, except for H_2_O_2_, which showed an opposite tendency compared to shoots, i.e., a decrease in tolerant lines.

***Chromium:*** Hexavalent chromium (Cr(VI)) is soluble in the pH range of natural waters, making it bioavailable, and it occurs in irrigation water. As it accumulates, it is toxic to terrestrial and aquatic organisms [49]. Christou et al. (2020) discovered that hexavalent Cr(VI) exposure at 5 and 10 mg L^−1^ K_2_Cr_2_O_7_ reduced biomass, leaf size, and the number of photosynthetic pigments while increasing lipid peroxidation, H_2_O_2_ and NO, SOD, and CAT activities, which were partially supported by MnSOD, FeSOD, Cu/ZnSOD, and CAT transcript levels [50], proline content, P5CS enzymatic activity, and corresponding P5CS and P5CR gene expression levels [50].

***Lead (Pb):*** Plant exposure to Pb leads to reduced growth, chlorosis, and a diminution in photosynthetic rates [51]. As reported by López et al. (2007), 40 mg/L of lead exposure resulted in a decrease in CAT activity but increased total amylase activity (TAA) in leaves [52]. Hattab et al. (2015) applied 0, 10, and 100 mM Pb for 2 and 7 days on alfalfa and measured a number of stress indicators [53]. Their results revealed a time- and dose-dependent accumulation of Pb that was more pronounced in roots than in leaves. A decrease in root glutathione (GSH) and homoglutathione (hGSH) levels also exhibited concentration dependency. The increased lipid peroxidation, glutathione reductase (GR), ascorbate peroxidase (APX), and superoxide dismutase (SOD) activities were complemented by the up-regulation of APX and SOD genes. Levels of heat shock proteins (HSP70 and HSP17.7) were found to be higher in alfalfa shoots, which suggests that lead phytotoxicity activated a cell-protection mechanism to cope with lead phytotoxicity [53].

***Mercury (Hg):*** Mercury causes toxic metal pollution in agricultural lands, results in metabolism disorders, and retards growth and development. Quantities of 4, 5, 10, 20, and 40 M Hg induced O_2_^−^ and H_2_O_2_ generation in alfalfa leaves and increased NADH-oxidase and lipoxygenase (LOX), APX, POD, and CAT activities [54,55]. Zhou et al. (2008) revealed that 24 h of 10 M HgCl_2_ strongly inhibited the activities of APX and GR in the roots of alfalfa [55] (Figure 3, Table 1).

**Table 1:** Plant part, applied heavy metal and concentration, affected physiological processes, accumulation in the plant, and the duration of treatment of HM-treated alfalfa plants.

Plant Part	Applied Heavy Metal and Concentration	Affected Physiological Processes	Accumulation in Plant	Duration of Treatment	Reference
Whole plant	Mo	decreased dry weight	346 ppm	21 days	[29]
Whole plant	Mn: 60 mg L^−1^	reduced dry weight by 20%	n.a.	36 days	[26]
Seed	Zn: 1.5–24 mM Pb: 1.5–24 mM	reduced germination rate	Zn: root: 490 mg kg^−1^; shoot: 180 mg kg^−1^ Pb: root: 1330 mg/kg, shoot: 300 mg kg^−1^	24 h	[25]
Roots	Zn: 0.038–50 µM Cd:0.45–141.2 µM	poor root development	Zn: 2700 mg kg^−1^ DW Cd: 1000 mg kg^−1^ DW	14 days	[24]
Roots	Mn	browning roots disrupted nutrient uptake	30–500 mg kg^−1^ DW	n.a.	[28]
Roots	Hg:10 μM	strong inhibition of APX and GR induced lipid oxidation	n.a.	0; 6; 12; 24; 48; 72 h	[55]
Roots	Mn: 500 µg g^−1^	decreased the growth of roots and shoots	1822 µg g^−1^ DW	49 days	[27]
Roots	Ni: 0; 50; 150; 250; 500 mg kg^−1^	increased POX, GST activities, increased MDA level up-regulated Prx1C, GST and PC genes	0.61; 1.96; 9.97; 11.68; 23.65 mg kg^−1^ DW respectively	60 days	[35]
Roots	Cu	constrained the reproduction of diazotroph bacteria (decrease N fixation)	n.a.	2 years	[36]
Roots	Cd: 3 and 5 mg kg^−1^	decreased root length and the uptake of K, Mg, Ca, Fe	600; 850 mg kg^−1^ DW respectively	7 days	[44]
Roots	As: 25–35 μM	nodulation decreased by 75%	n.a.	3; 6; 10; 28 days	[43]
Root	Cd: 1 mM	decreased soluble proteins decreased enzymatic activity of CAT, APX, SOD enhanced electrolyte leakage, iron chelate reductase activity, total phytochelatin, citrate and malate levels increased H_2_O_2_ content up-regulated three Fe-related genes: MsIRT1, MsNramp1, MsFRO1	root: 10 mg kg^−1^ DW	7 days	[47]
Root	Cd: 0–40 μM	tolerant lines: increased cadmium content, increased dry weight tolerant lines: increased percentage of relative germination tolerant lines: reduced amount of hydrolyzed starch tolerant lines: lower level of lipid peroxidation and the loss of plasma membrane integrity tolerant lines: unchanged SOD activity tolerant lines: lower of H_2_O_2_	600–1700 mg kg^−1^ DW in tolerant cultivars and 600–1450 mg kg^−1^ DW in non-tolerant cultivars	48; 72; 96 h	[48]
Stem	Cu: at high availability in soil	influence the cell wall properties (decreased quality) reduced level of ferritin, redox status of the cell	n.a.		[37]
Root, Shoots	Pb: 0; 10; 100	decreased GSH and hGSH levels increased the lipid peroxidation level, GR, APX, SOD activity and HSPs up-regulated APX and SOD genes	root: 766.66 mg kg^−1^ DW shoot: 385.67 mg kg^−1^ DW	2 and 7 days	[53]
Shoots	Ni: 50; 150; 250; 500 mg kg^−1^	increased POX, GST activities increased MDA level up-regulated Prx1C, GST and PCs genes	1.58; 8.92; 22.64; 32.84; 75.2 mg kg^−1^ DW respectively	60 days	[35]
Shoots	Mn: 500 µg g^−1^	decreased growth of roots and shoots	753 µg g^−1^ DW	49 days	[27]
Shoots	Cd: 3 and 5 mg kg^−1^	decreased shoot length	600; 110 mg kg^−1^ DW respectively	7 days	[44]
Shoots	Cd: 1 mM	decreased the level of total soluble proteins and enhanced electrolyte leakage, iron chelate reductase activity, total phytochelatin, citrate and malate levels decreased enzymatic activity of CAT, APX, SOD increase H_2_O_2_ content up-regulated three Fe-related genes (MsIRT1, MsNramp1, MsFRO1)	1.4 mg kg^−1^ DW	7 days	[47]
Shoots	Cd: 0–40 μM	tolerant lines: increased cadmium content, increased dry weight tolerant lines: increased percentage of relative germination tolerant lines: reduced amount of hydrolyzed starch tolerant lines: unchanged SOD activity tolerant cultivars: higher level of H_2_O_2_	25–31 mg kg^−1^ DW in tolerant and non-tolerant cultivars	48; 72; 96 h	[48]
Leaves	Cr: 0.05; 0.5; 1; 5; 10 mg L^−1^	reduced biomass, leaf size and amounts of photosynthetic pigments increased the level of lipid peroxidation, H_2_O_2_, NO, SOD and CAT activity, prolin content, P5CS enzyme activity, and expression of GST7, GST17, P5CS, P5CR MnSOD, FeSOD, Cu/ZnSOD, and CAT genes	2.5; 2.8; 5; 8; 16 mg kg^−1^ DW	59 days	[50]
Root Cotyledon Leaves	Pb	reduced growth, chlorosis and lowered the photosynthesis rate	Root: 25,500 mg L^−1^ DW Cotyledon: 300 mg L^−1^ DW Leaves: 29 mg L^−1^ DW	50 days	[51]
Leaves	Pb: 40 mg L^−1^	decreased CAT activity and increased TTA	n.a.	10 days	[52]
Leaves	Hg: 1; 5; 10; 20; 40 µM	induced H_2_O_2_, O_2_^−^, and increased NADH-oxidase, LOX, APX, POD, CAT activity	n.a.	0; 6; 12; 24; 48; 72 h	[54]
Leaves	Zn: 4–7.3 mM	chlorotic symptoms with necrotic spots	Zn: shoot: 300 mg kg^−1^ DW Cd: shoot: 40 mg kg^−1^ DW	14 days	[24]
Leaves	Mn	interveinal chlorosis in young leaves necrotic spots in mature leaves generally triggered oxidative stress symptoms	n.a.	n.a.	[28]

## 5. Phytoremediation

Phytoremediation is the process of using plants to remove heavy metals and other pollutants from soil or nutrient media. The success of phytoremediation is based on a better understanding of the effects of heavy metals on plants. As non-essential heavy metals cannot be incorporated into plant metabolism, it is particularly important to prevent heavy metals from exerting their harmful effects. One possible way to extract HMs from contaminated soil is to have them taken up by and concentrated in an accumulator species, and then removed from the site [56]. The resulting biomass can potentially be used to recover the metal if it is economically viable [57]. Only about 400 plant species worldwide are known to be able to hyperaccumulate heavy metals. The accumulative metals are cadmium, chromium, copper, manganese, nickel, and zinc [56]. Most of the accumulating plants are members of the *Brassicaceae* family, but plant species that are symbiotic with nitrogen-fixing bacteria, such as alfalfa, have the potential for phytoremediation. However, in these plants, metal fixation occurs mainly in the root zone, the site of symbiosis, and extraction from the soil is an additional challenge. Phytoremediation is successful if the plant metabolism remains intact during accumulation. The literature on heavy metals shows that most species capable of hyperaccumulation do not develop large leaf masses or do not do so at large scales [57]. It may be an important task to study the responses of species under agricultural cultivation to heavy metal stress because they may be potential species or varieties that can be selected for later use as efficient bioaccumulators.

## 6. Heavy Metal Alleviation Possibilities

### 6.1. Role of Fungi in Mitigating Heavy Metal Stress

In the majority of studies, fungi were identified as stress-relieving agents. In the root zone, it is noted that the treated fungi form a symbiotic relationship with the plant. Mycorrhiza describes this sort of interaction between fungi and plant roots. This interaction is symbiotic because, while the fungi aid the plant in absorbing nutrients such as phosphorus (P) and nitrogen (N), the plant also provides the fungi with numerous organic compounds, most notably carbon from photosynthesis. The subsequent partnership can be mutually beneficial [58]. This form of mycorrhiza is known as arbuscular mycorrhiza (AM). The fungi of AM are capable of penetrating the cortical cells of plant roots, so creating a symbiotic relationship [59]; meanwhile, the hyphae penetrate the surrounding soil, achieving twice as much soil volume as plants lacking AM [60,61]. Thus, nutrients with low mobility, such as P and/or zinc, are made available to plants. Therefore, the primary function of AM symbiosis is to enhance plant absorption [61].

For arsenic, cadmium, chromium, lead, mercury, and nickel, HM tolerance and accumulation capability of arbuscular mycorrhizal (AM) fungi have been demonstrated [24,35,62,63]. In addition, mycorrhized plants are known to accumulate the most HMs in their roots [64,65]. Several plant mechanisms have been identified as aiding in the alleviation of stress caused by HMs. Examples include transit to and storage in root cell vacuoles, extracellular chelation in the root, and binding of HMs to the cell wall of rhizoderm cells [66]. AM fungi can contribute to HM traffic in two ways. First, they can absorb and transport huge quantities of HMs to the roots of the plant, raising the concentration in the root; second, they can neutralize the HMs in their hyphae or soil. These mechanisms depend on the species of fungi and soil parameters [66]. To reduce HM stress, the fungus employs plant-like processes, including HM immobilization with different compounds, binding to the chitin cell wall, and chelation. Fungi create and release into the soil a glycoprotein called glomalin, which is one of the most important known compounds. This molecule in the soil binds HMs [66].

*Glomus* species have a greater potential for binding HM than other microorganisms. HM-tolerant ecotypes (separated from HM-contaminated soils) also have the maximum binding capacity [67]. *Glomus mosseae* samples isolated from HM-contaminated soils are much more tolerant of increased Cd concentrations than those isolated from soils devoid of HM. This shows that the fungus has adapted to high levels of HM, making it likely that HM-tolerant ecotypes exist in nature [68].

The symbiotic interactions of AM fungus are well-known in our natural world, but less so in agriculture. Numerous widespread agricultural practices, such as the use of fertilizer, biocides, and monoculture, are damaging to AM fungus. Only organic production methods may be able to remedy the lack of AM fungus in production ecosystems caused by these activities. These agricultural methods are less harmful to AM fungi because they avoid the use of water-soluble fertilizers and biocides, and they typically employ crop rotation. In a 2006 study, Gosling and colleagues evaluated the possibilities of integrating the usage of AM fungi into conventional farming systems and the most effective methods for exploiting the benefits of AM fungi in such a management system [69].

Wang et al. (2012) conducted an experiment to determine the effects of AM fungus on Cd-contaminated soil-grown alfalfa. In the experiment, the fungus *Glomus intraradices* was used, and the subcellular distribution and chemical forms of cadmium were the primary emphasis. On the 25th day following planting, the alfalfa roots were found to be heavily invaded by the fungus. Consequently, the biomass of plants inoculated with AM fungi was 1.7 times more than the biomass of plants not infected with AM fungi. Without inoculation, alfalfa shoots contained about three times more Cd. After treatment, it was also found that the majority of Cd was concentrated in the roots. These experimental results may provide a solid foundation for the future application of AM fungi in agricultural production [70]. Moreover, this result suggests that this approach may be useful for the use of alfalfa plants under HM contamination as forage, as it binds a substantial quantity of cadmium in the root zone.

Similar results were found by Motaharpoor et al. (2019), who evaluated the influence of an AM fungus *Rhizophagus irregularis* inoculation on cadmium accumulation in alfalfa and HM chelating agents, phytochelatins [71], in a greenhouse experiment. Following Cd exposure, the gene expression of phytochelatin synthase is activated and phytochelatins are synthesized from glutathione-based peptides. Phytochelatins are complex, amino acid-containing peptides comprising glutamine, cysteine, and glycine, which form different complexes with HMs so that the thiol group of the cysteine forms a chelate with the HM ion, preventing cadmium ions from exerting their damaging effects in the cytoplasm [72]. Phytochelatins bind cadmium and release it into the vacuole via a Cd^2+^/H^+^ antiport mechanism [73]. Under both stressed and non-stressed situations, the fungal symbiosis enhances alfalfa biomass [72,73]. In addition, the data demonstrated that *Rhizphagus irregularis* greatly reduces the presence of cadmium in the shoots and that the fungus significantly activates the chelating chemicals. This may be a way to eliminate HMs from alfalfa production, at least in shoots [71]. This study’s findings suggest a positive future for agricultural output, since they confirm the fundamental premise that HM concentrations should be lowered in the above-ground environment.

Zaefarian et al. (2011) conducted two studies involving various types of fungi. In the first experiment, they utilized *Glomus mosseae*, *Glomus etanicatum*, *Glomus intraradices*, *Glomus fasciculatum*, and *Gigaspora hartiga*, and combinations of these strains. In this experiment, alfalfa’s nutrient uptake was analyzed, and the results indicated that the strains were successful in aiding alfalfa’s nutritional uptake. Subsequently, alfalfa seeds were sown in soil contaminated with Cd, Co, and Pb, and the soil was inoculated with the *Glomus mosseae* strain. It was determined that AM fungal inoculation had a good influence on alfalfa growth [74] based on a number of findings.

Dark septate endophytic (DSE) fungi are a subgroup of anamorphic *Ascomycetes* fungi. Similar to AM fungi, DSE fungi can create mutualistic connections with plant roots [75]. Hou et al. (2020) conducted an experiment on DSE fungi isolated from cadmium-contaminated soil in an effort to determine the viability of using DSE fungi in the growth of diverse crops, thereby contributing to the HM removal strategies for polluted soils [76]. In the experiment, *Acrocalymma vagum* and *Scytalidium lignicola* were utilized. It was observed that inoculation with *S. lignicola* greatly raised the organic carbon content of alfalfa and enhanced the accumulation of cadmium in the roots of the examined plants. These findings suggest that the administration of DSE fungi boosted root growth, promoted plant survival under cadmium stress, and greatly increased shoot biomass [76].

The impacts of AM fungi (*Glomus intraradices*) on the development, photosynthetic processes, and protein content of alfalfa seedlings grown in lead (Pb)-contaminated settings were analyzed by Persian researchers. The results demonstrated that Pb stress inhibited plant development and impeded photosynthesis, but AM fungal inoculation mitigated these detrimental effects. In addition, the treatment of the fungus decreased the amount of lead in the plant’s shoots and roots. The results indicate that *Glomus intraradices* may mitigate the toxicity of lead in alfalfa plants [77].

Based on the results of the aforementioned studies, it appears that the use of individual fungal species can be an effective method in forage alfalfa plantations where plants are under HM stress; however, in some studies, fungi were applied in combination with other active substances, which may have improved the results.

### 6.2. Combination of Organic Substances with Fungi

Biochar is a substance that is produced by the thermochemical conversion of biomass in oxygen-poor environments. It is mainly used for soil amendment purposes [78]. Zhang et al. (2019) investigated the results of combining biochar and AM fungi on alfalfa grown in Cd-contaminated soil [12]. In this experiment, the researchers observed the effects of biochar, AM fungus inoculation, and cadmium on the growth and yield of alfalfa. The presence of Cd in the soil significantly reduced the biomass of alfalfa, and the nitrogen (N), phosphorus (P), calcium (Ca), and magnesium (Mg) contents in shoots and roots were also greatly reduced. The addition of AM fungi significantly increased N and P contents in the plants. Moreover, biochar significantly increased alfalfa biomass weight and N, P, K, and Ca contents. The combination of AM fungi and biochar resulted in the lowest Cd concentration and the highest nitrogen and phosphorus contents in the shoots. From these results, it can be concluded that the combined application of AM fungi and biochar helped alfalfa nutrient uptake and had a positive effect on its growth in cadmium-contaminated soil [79]. Similar results and conclusions were obtained by Anas Raklami et al. (2020) [80]. They conducted an experiment to investigate the effects of compost and AM fungi on alfalfa grown in an environment contaminated with Cd and Zn. Compost is a humus-like material, formed by the fermentation through biological oxidation of green waste, usually from plant and food sources, used to improve soil conditions. In the experiment of Raklami et al. (2020) [80], green waste collected from the city of Marrakesh was composted in a municipal composter. After three months of composting, the compost had a pH value of 7.86, a carbon content of 30.65%, a nitrogen content of 2.19%, and a crude ash content of 49%. Cd, Pb, and other HMs were not detected in the sample. The combined application of AM fungus (*Rhizophagus irregularis*) and compost resulted in a significant increase in the biomass produced by the alfalfa. Furthermore, the treatment increased the conductivity of the stomata, increased the sugar and chlorophyll contents of the plant, and reduced the accumulation of HMs in the plant [80], providing a new opportunity to help alleviate HM stress on alfalfa farms.

### 6.3. Plant Growth-Promoting Rhizobacteria (PGPR) Were Applied to Alfalfa Plants

The study presented above yielded successful results in mitigating the stress effects of HM pollution using AM fungal strains, sometimes in combination with bacteria. Raklami et al. (2020) [80] conducted a rhizobacterial inoculation experiment. This symbiotic coupling resembled the coupling by fungi that was discussed previously [70]. HM contamination demonstrated detrimental impacts on the chlorophyll content, fluorescence, and gas exchange of alfalfa grown in HM-contaminated soil, where metal-resistant bacterial inoculants decreased metal stress. Even in samples polluted with HMs, the microorganisms were able to successfully colonize plant roots, hence promoting plant development. These findings demonstrated that metal-resistant rhizobacteria may play a crucial role in the clean-up of metal contamination. Furthermore, inoculated legume crops were still able to be safely cultivated since the inoculation lowered the concentration of HMs that accumulated in the plant’s above-ground portion [80]. The experimental results reported above support the concept that bacteria can be employed to efficiently reduce HM stress by sequestering HMs in the plant’s underground tissues. Tirry et al. (2021) investigated the unfavorable effects of Cr(VI) [81]. It was anticipated that rhizobacteria would assist in enhancing plant phytoremediation. The results demonstrated that the 27 Cr(VI)-resistant rhizobacteria were advantageous for alfalfa growth: shoot and root mass rose and oxidative stress decreased [81].

Chen et al. (2018) conducted a study to identify a way to reduce copper stress in alfalfa. In their experiment, they investigated the impact of the bacterial species *Sinorhizobium meliloti* on alfalfa seedling growth under copper stress [82]. The results demonstrated that bacterial inoculation mitigated the effect of Cu-induced growth inhibition and improved nitrogen uptake by the seedlings, consequently raising the nitrogen concentration in the plants. Inoculated seedlings exhibited considerably greater Cu absorption than uninoculated ones. This technology can be utilized effectively to improve phytoremediation’s effectiveness. Greater Cu accumulation was seen in roots compared to shoots, and inoculation decreased Cu stress-induced lipid peroxidation and reactive oxygen generation. Overall, bacterial inoculation had a favorable impact on alfalfa growth and critical functions in Cu-contaminated soil [82].

Another experiment examined the growth of alfalfa seedlings in multiple types of HM-contaminated soil in the presence of various bacterial strains. Alfalfa seedlings were grown in HM-contaminated soil with bacteria in an effort to identify the most effective bacterial strains. Using bacterial strains capable of making indole-3-acetic acid, an auxin-type plant hormone, the results showed that seven of the strains encouraged shoot growth in alfalfa seedlings. In addition, after alfalfa plant inoculation, significant Cd and Pb accumulation was seen in the rhizosphere. Two bacterial strains were advantageous for alfalfa growth and HM fixation, as shown by the study’s findings. *Bacillus filamentosus* and *Bacillus cereus* are the two bacterial strains in this pair. Based on the data, it can be inferred that these two bacterial strains may offer exceptional potential for alfalfa production in HM-contaminated areas, contributing to the development of phytoremediation procedures [25].

The results of the tests provided above indicate that the use of diverse bacterial strains in alfalfa, in addition to aiding in the plant’s uptake of nutrients, may be a suitable tool for the control of HM stress in a manner comparable to the use of AM fungus. Ghnaya et al. (2015) [7] also treated Cd-contaminated soil-grown alfalfa with bacteria (*Sinorhizobium meliloti*). The primary criterion for selecting this bacterium was its ability to tolerate metals. Sixty days were spent cultivating inoculated and uninoculated plants with bacterial strains. At the end of the period during which the plants were grown, it was noted that inoculation alleviated the formation of numerous Cd toxicity symptoms in the inoculated seedlings. These symptoms manifested on the branches of non-inoculated plants. In addition, bacterial injection enhanced biomass output and facilitated nutrient intake. A quantity of 50 mg of Cd was found in 1 kg of soil during the experiment. The amount of Cd recovered from the shoots of infected plants was 58 g, whereas it was 178 g for non-inoculated plants [7]. The amounts of Cd content indicate that the application of bacterial inoculation significantly reduced the amount of Cd in shoots; therefore, it can be inferred that this method is capable of reducing the negative effects of HM contamination on alfalfa agriculture.

Gan and colleagues (2020) [83] employed vanadium (V) in an experiment in which they produced alfalfa in soil contaminated with varying quantities and evaluated the effects after bacterial application. In the experiment, soil samples with varying levels of contamination were exposed to a vanadium-resistant strain of the bacterium *Arthrobacter*. At low levels of contamination, alfalfa shoot and root mass rose significantly following inoculation compared to the uninoculated control. The shoot and root dry weight rose at moderate levels of pollution. However, at high vanadium contamination levels, non-inoculated plants were destroyed by vanadium, whereas inoculated plants resisted the harmful effects of metal pollution. These findings indicate that plant-microbe co-existence may be a viable solution for vanadium-contaminated soil. The usage of this metal-resistant bacterial strain has a good effect on the development of vanadium-contaminated plants, the researchers determined. This indicates that metal-resistant bacteria sprayed in metal-contaminated areas have a positive effect on plant physiological processes [83].

These findings demonstrate that the inoculation of alfalfa with PGPR bacteria enhances the retention of the majority of the HM in the root zone; as a result, the plant’s above-ground portions are safe for ongoing use, including as pasture.

### 6.4. Combining Fungi and Bacteria

Some researchers applied combinations of microorganisms of fungal and bacterial origin. Tabande et al. (2021) [8] investigated the negative effects of zinc oxide nanoparticles and ways to mitigate these effects in alfalfa seedlings. Fertilizers made from zinc oxide nanoparticles are among the most commonly used fertilizers today. Their use generally has a positive effect on plant growth, but their excessive presence in the soil has adverse effects when they accumulate in high levels in plant tissues. In this study, alfalfa seedlings were planted in soil contaminated with zinc oxide nanoparticles and treated with rhizobacteria and fungal inoculations. They used strains of the bacterium *Sinorhizobium meliloti* and the fungus *Serendipita indica* in the inoculation. The zinc oxide nanoparticles were added at different concentrations to the soil used for planting alfalfa seedlings. The results obtained showed that inoculation with the *Serendipita indica* strain minimized the growth inhibition stress effects caused by zinc oxide nanoparticles by enhancing the growth rate and binding zinc in the root, resulting in reduced zinc concentration in the shoots. Inoculation with the bacterium *Sinorhizobium meliloti* increased shoot dry weight. The optimal effects were observed when the two strains of bacteria were used together. In samples contaminated with different concentrations of zinc oxide nanoparticles, the translocation of zinc from roots to shoots was reduced by about 60.2% and 44.3%, respectively. The oxidative stress caused by zinc was reduced by the application of *S. indica* fungus by increasing the activity of catalase and peroxidase enzymes. The results of this study indicate that the use of the endophytic fungus *Serendipita indica* and the bacterium *Sinorhizobium meliloti* in crop production has the potential to contribute to the improvement of feed HM tolerance and food safety factors for human consumption by guaranteeing a reduction in zinc content in the above-ground parts of plants [8].

Wang et al. (2021) [84] conducted an experiment showing that rhizobacteria and AM fungi are beneficial for legume crops growing in HM-contaminated soil. Microbiomes contribute to mitigating the toxicity of plants induced by HM stress. In the study, the researchers investigated the effect of inoculating rhizobacteria and AM fungi on alfalfa’s resistance to cadmium (Cd) stress. The results showed that separately applied rhizobacterial and AM fungal inoculation significantly increased the Cd stress tolerance of alfalfa, with some increase in plant biomass. The combination of bacteria and AM fungus inoculation resulted in the greatest overall improvement. The enhanced condition of the plants was evidenced by the reduction in Cd stress-induced lipid peroxidation and oxidative stress. Co-inoculation of the two microorganisms significantly changed the structure of the microbial community found in the rhizosphere. The diversity of fungal communities decreased, while the diversity of bacterial communities increased. In addition, the researchers observed that the combined application of AM fungi and rhizobacteria increased the nutrient uptake capacity of alfalfa in the rhizosphere and reduced the release of carbon from photosynthetic activity into the soil. The results show that inoculation with co-applied microorganisms can support plants in coping with Cd-induced stress effects. Furthermore, the study will help to understand the microbial processes that take place in the rhizosphere, providing a better understanding of how bacterial and fungal communities in the rhizosphere function [83]. The two studies presented above provide adequate support for an earlier finding that the combined use of rhizobacteria and AM fungi may be a suitable way to grow alfalfa in HM contaminated environments, thereby improving phytoremediation (Table 2).

In an early study of EI-Kherbawy et al. (1989), the researchers investigated the relationships between high Cd and Zn soil pH, the effects of bacterial and fungal inoculations applied, and the development of planted alfalfa seedlings [4]. Alfalfa seedlings were planted in soil samples of different pH values and treated with rhizobacterial inoculation and vesicular arbuscular mycorrhizal fungal inoculation. In soils with pH 4.3 and 5.3, the alfalfa seedlings did not survive due to soil acidity and HM content in the soil. In higher-pH soils, an increase in alfalfa biomass was observed in both bacterial and AM fungal inoculations, and shoot nitrogen and phosphorus concentrations were also greatly increased. The greatest physiological effect was obtained when AM fungi and rhizobacteria were applied together. This resulted in the most significant decrease in HM concentrations in the above-ground parts of the plants and the greatest increase in biomass [4].

**Figure 4:**
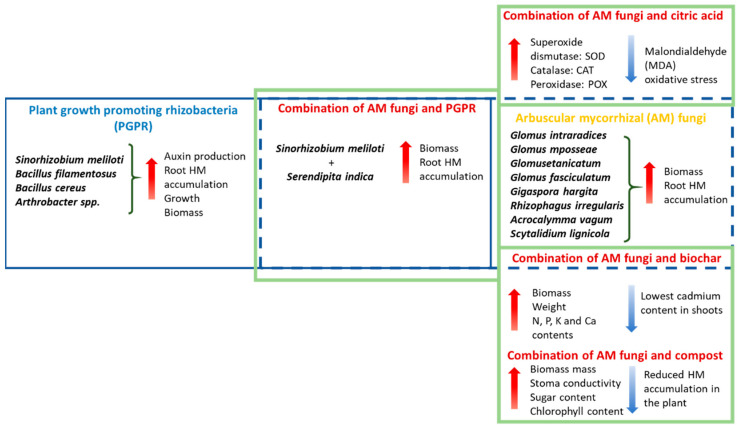
Single and combined application of AM fungi, bacteria, and organic substances, and their investigated effect on plant metabolism.

The results of this study also show that the combined use of bacteria and AM fungi can be effective in improving phytoremediation processes and, in turn, HM removal processes in alfalfa plantations produced by agriculture. These studies are useful for stress relief and the results are valuable, but can only be exploited if appropriate tools exist to introduce these organisms into the environment. It is the responsibility of pesticide researchers and pesticide companies to materialize these organisms and then guarantee their safe use so that the studies presented above can benefit all producers who suffer from the effects of HM pollution (Figure 4).

### 6.5. The Application of Salicylic Acid

One of the most important endogenous plant hormones is salicylic acid. It is essential to multiple plant regulatory, physiological, and metabolic processes [85]. It is one of the most important signal transduction molecules and plays a crucial role in mitigating both abiotic and biotic stresses. The application of optimal concentrations of salicylic acid also increases the tolerance of plants to HM pollution [86].

The objective of the research described below was to gain insight into the response of aluminum (Al)-contaminated plants to salicylic acid treatment, thereby reducing Al toxicity. It was observed that the Al content in the leaves increased over time, resulting in leaf damage and chloroplast deformation. Due to the toxicity of Al, the double membrane of chloroplasts in the leaves was compromised, starch granules vanished, and additional damage was observed. Foliar spraying of salicylic acid decreased Al content in roots and leaves and alleviated the previously mentioned chloroplast symptoms. Externally, chloroplasts had a flat, ellipsoidal shape, whereas, internally, thylakoids were tightly packed and regularly spaced, and intact starch granules were observed. In comparison to untreated plants, salicylic acid treatment led to a substantial increase in the above-ground biomass of treated plants. The results demonstrated that salicylic acid mitigates the harmful effects of Al toxicity by increasing light-harvesting efficiency and thereby promoting electron transport [86]. Salicylic acid may play an important role in future research as it functions to preserve and improve photosynthetic functions under Al stress [87]. In alfalfa seedlings treated with salicylic acid, the inhibition of shoot and root growth caused by Cd was reduced. Potassium (K), magnesium (Mg), calcium (Ca), and iron (Fe) levels decreased significantly in both shoots and roots as a result of Cd contamination. After treatment with salicylic acid, these nutrients began to accumulate in the plant. In addition, a rise in total biomass was noted as a result of the treatment [44].

This experiment also demonstrates that salicylic acid can be effective for managing stress in Cd-contaminated alfalfa crops. In the next study to be presented, researchers examined the effects of salicylic acid in a mercury-contaminated environment (Hg). They discovered that salicylic acid significantly increased root elongation, which had been severely inhibited by mercury. These findings suggest that microorganism combinations may also be useful in alfalfa cultivation for agricultural purposes. These researchers demonstrated in a previous study that Hg causes severe oxidative damage to both the roots and leaves of alfalfa plants. However, they demonstrated in this study that salicylic acid mitigated Hg-induced oxidative damage both in roots and leaves. In addition, the experiment demonstrated that salicylic acid was effective at protecting the cell membrane from Hg-induced oxidizing substances. In conclusion, the researchers demonstrated that salicylic acid can regulate Hg-induced oxidative stress in alfalfa roots [54].

### 6.6. Organic Acids

***Citric acid:*** Application of exogenous CA results in improved growth and yield in crops exposed to abiotic stressors [88]. In this experiment, the researchers investigated the effects of citric acid and the surfactant Tween^®^ 80 (polyethylene glycol sorbitan monooleate) on the development of alfalfa plants in HM-contaminated environments.

Additionally, they investigated the possibility of utilizing these compounds to enhance phytoremediation processes. The results demonstrated that the plants can tolerate HM pollution and begin to grow. After 90 days of experimentation, shoot and root biomass increased as predicted, and there was negligible plant mortality in the samples tested. This observation demonstrates that alfalfa can tolerate HMs to a limited extent but that, in the presence of HMs, both shoot and root mass increase. The soil amendments used in the experiment did not significantly increase the concentrations of metals in the plants or the number of microorganisms in the soil. After 30 days, however, the application of citric acid and Tween^®^ 80 increased the number of microorganisms in the soil by 2.4-fold. In addition, the lipase enzyme activity increased by 5.3-fold. This experiment provides evidence of and a response to the observation that alfalfa can tolerate the presence of HMs in environments contaminated by multiple HMs, such that the effect of Tween^®^ 80 and citric acid increases the microbial population in the rhizosphere. This experiment may have future phytoremediation applications [3], and confirms that the use of citric acid may be a viable technique for alfalfa cultivation in metal-contaminated regions.

***Ascorbic acid:*** Exogenous ascorbic acid induced the up-regulation of alfalfa heme oxygenase-1 (HO-1) transcript and decreased oxidative stress in cadmium-contaminated alfalfa seedlings. Moreover, cadmium concentration and the rate of growth inhibition caused by Cd were reduced [89].

### 6.7. Combined Use of Citric Acid and AM Fungal Strains

To examine the combined effects of citric acid and AM fungal inoculation in vanadium (V)-contaminated soil, a greenhouse pot experiment was conducted. The measurements revealed that the accumulation of HM in plant tissues led to a significant decrease in plant biomass and root mycorrhizal colonization, and a substantial increase in the accumulation of V in plant tissues. An increase in phosphorus uptake was also observed as a result of citric acid treatment. The AM fungus inoculation increased the dry weight and phosphorus content of the plants’ shoots and roots. The combined treatments significantly contributed to a decrease in malondialdehyde (MDA) content, indicating a reduction in oxidative stress, and greater antioxidant activity (SOD, POD, and CAT) was measured in the leaves. This suggests that the combination of substances may stimulate alfalfa’s antioxidant response and promote plant growth. The symbiosis of citric acid and AM fungus provides a new remediation strategy for alfalfa in vanadium-contaminated environments [90].

### 6.8. Further Applications

***Heme-oxygenase:*** In the next two studies, the hem-oxygenase enzyme was applied to alfalfa plants exposed to an HM-contaminated environment. In the first study, researchers examined the development of alfalfa grown in a Cd-contaminated environment [91]. Severe oxidative damage was observed in the roots of Cd-contaminated seedlings, but heme oxygenase prevented this toxic response. In addition to a decrease in lipid peroxidation, growth inhibition, and Cd accumulation, a decrease in lipid peroxidation was also observed. Overall, the data indicate that heme oxygenase offers substantial protection against Cd-induced damage [87]. Moreover, Cui et al. (2013) examined the effects of Al and hem-oxygenase on alfalfa plants [92]. In summary, the researchers concluded that heme oxygenase plays a significant role in mitigating Al-induced stress effects. The researchers’ intervention had similar beneficial effects to those described in the previous study. Reduced oxidative stress, decreased metal content in the plant, and improved growth parameters were found [92].

***Flavodoxin protein:*** Flavodoxin is an electron transport protein that plays a role in the response to oxidative stress. Shvaleva et al. (2009) investigated the responses of rhizobacteria and alfalfa plants in Cd-contaminated environments in the presence of flavodoxin protein, which was positive for nitrogen fixation by alfalfa plants in Cd-contaminated environments [89]. Flavodoxin significantly decreased the amount of Cd in the plant and induced structural and ultrastructural changes in root nodules. These nodules were likely formed as a defense mechanism in response to the overexpression of flavodoxin. These findings imply that flavodoxin can be used as a biotechnological treatment to enhance the symbiotic performance of alfalfa and other legume life processes in cadmium-contaminated soil [93].

***Silicon****:* The addition of Si to Cd-exposed plants led to significant improvements in morpho-physiological traits, an increase in total protein content, and improved membrane stability, indicating that Si may play a significant role in mitigating Cd-induced stress effects. The Si treatment decreased the HM content in both shoots and roots and increased plant biomass. This study provides evidence of the beneficial effects of Si treatment on Cd-contaminated alfalfa, and its findings can be used to improve phytoremediation [47].

***Gases*** (CO, H_2_, CH_4_, H_2_S, and NO): In a study by Han et al. (2007), the effects of dissolved carbon monoxide (CO) were investigated on HM-contaminated alfalfa seedlings [94]. Different concentrations of CO had varying beneficial effects on alfalfa development. The results indicated that the addition of exogenous CO to the alfalfa production medium protected the plant from HM-induced oxidative damage, thereby reducing the plant’s negative effects [94].

In a study by Dai et al. (2016), the experiment was conducted using hydrogen (H_2_) in the form of aqueous solution, and the proteins induced by the treatment were evaluated and categorized [95]. The experiment showed that H_2_ reduced Cd-induced toxicity in alfalfa seedlings, in addition to oxidative stress and lipid peroxidation effects. The plant’s metabolism and nutrient uptake were enhanced [95].

CH_4_ pretreatment significantly alleviated the negative effects of copper stress, including seed germination and seedling growth inhibition. Overall, the plant’s Cu accumulation was diminished. It was also observed that sugar content increased and copper-induced oxidative stress decreased. The results demonstrated that CH_4_ treatment mitigates seed germination inhibition and reduces toxic effects on the plant [96].

Another study presented in the gases group examines the effects of the combined application of hydrogen sulfide (H_2_S) and nitric oxide (NO) on Cd-contaminated forage alfalfa seedlings (Cd). In general, the application of the treatment decreased Cd toxicity. This conclusion was supported by a significant improvement in seedling growth parameters and a decrease in lipid peroxidation. In addition, the results revealed that Cd accumulation in the plant was reduced. Overall, these results suggested that the gases utilized in this experiment may be suitable for supporting alfalfa grown in HM-contaminated soils and mitigating the toxicity of HM [10].

## 7. Potential Directions of HM Stress Research in Alfalfa

### 7.1. Element Effects

**Figure 5:**
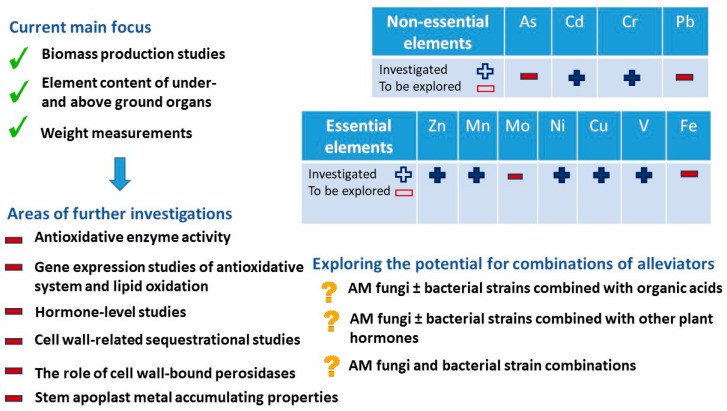
Current status and developmental possibilities of HM alleviation investigations.

The literature review shows that a number of essential and non-essential heavy metals have been studied in detail in alfalfa, but there are elements in both metal groups that have not yet been studied or have not been studied in sufficient depth. The literature overview indicates that, among the essential elements, these are molybdenum and iron, and, among the non-essential heavy metals, arsenic and lead require further investigation (Figure 5).

### 7.2. Cell Wall Processes

In addition, the literature review revealed that many HM stress detoxification processes in alfalfa, including those that have been studied extensively in other crops, have not yet been investigated in detail. These include the selectivity processes in various tissues. It is well established that both the root and shoot cell wall regions are capable of sequestering significant amounts of HMs, but only a few works on this topic have been found in alfalfa [76,95,97]. In alfalfa, cadmium lowered the protein level of G6PDH leading to a decreased amount of NADPH, which leads to impeded physiological mechanisms that require NADPH as a reductant. It is known that cadmium induces oxidative stress and changes in soluble and ionically bound cell wall peroxidase activities in roots of *Brassica juncea* (L.) *czern* seedlings; however, similar processes in alfalfa are yet to be elucidated [98].

### 7.3. Complexity of Research Works

Studies on the consequences of HM stress in alfalfa have shown a wide variation in methodology. In many cases, only a few analytical methods are used, such as dry weight, germination rate, and organ development [24,25,26,27,28,29,43,44]. However, in some cases, a wider range of studies has been conducted, spanning from changes in gene expression and enzyme activity changes to changes in metabolite levels [35,38,47,53,54,99], which provides a broader perspective of the effects of HM stress alleviator agents and, consequently, a deeper understanding of stress.

The fungal and bacterial strains have a biomass-enhancing effect when applied individually or in combination, and also help to retain HMs in the roots, according to studies. However, the combination studies did not always address how beneficial the combination itself is, or how the interaction between the studied organisms affects HM detoxification. Exploring the potential of combinations is an additional task for researchers, particularly in relation to the objective of reducing the presence of heavy metals. Indeed, if the aim is to reduce the heavy metal content of above-ground organs, the number and combination of materials that can be used are very high and varied. However, to purify soil-polluting HMs for phytoremediation purposes, even the opposite mechanisms are desired: the heavy metals should be translocated in large quantities from the soil to above ground and efficiently excreted by the plant.

## 8. Conclusions

It is evident from the results of the reviewed studies that HM pollution poses significant risks to alfalfa production. The stress effects of HMs reduce the crop’s biomass, thereby reducing the amount of forage, which has negative effects on production. In addition, the health effects of consuming HM-contaminated crops are a significant concern. Some HMs are extremely toxic, but high concentrations of less-toxic HMs also pose significant health risks.

The application of AM fungal and bacterial culture studies provides an opportunity for alfalfa growers to reduce the HM content in the plants, thereby reducing the additional health risks of feed produced from the crop. It is assumed that the results of these studies provide evidence for the use of compounds with beneficial effects.

In this review, the presented the literature provides guidance for the mitigation of HM stress in alfalfa (*Medicago sativa* L.) production. The purpose of the study was to identify methods for reducing the concentration of HMs in the plant, so that its continued use will be safer. From the perspective of agricultural production, this entails reducing the quantity of HMs accumulated in the shoots. The results demonstrated the existence of numerous compounds/organisms capable of reducing HM toxicity in alfalfa plants: AM fungi and various bacterial strains, organic acids, an enzyme, flavodoxin protein, certain gases, and silicon all had positive effects on the alleviation of the effects of HM on alfalfa, as evidenced by a reduction in oxidative stress, an increase in photosynthetic activity, an increase in biomass, improved plant growth parameters, and a decrease in HM content in both leaves and leaves and shoots. In the future, it will be beneficial to search for potential combinations based on the mode of action of those that have already been investigated and to provide more in-depth information through gene expression and metabolic studies.

## Figures and Tables

**Table 2:** Plant part, applied fungi and/or bacteria, affected physiological processes and the duration of treatment of HM-treated alfalfa plants.

Plant Part	Applied Heavy Metal and Concentration	Applied Stress Alleviator	Affected Physiological Processes	Duration of Experiment	Reference
shoots and roots biomass	Cd 0.5, 5, and 20 mg kg^−1^	*Glomus intraradices* AMF	growth, heavy metal uptake	80 days	[9]
shoots and roots biomass	Cd 100 mg kg^−1^	*Rhizophagus irregularis* AMF	shoots and roots dry weight, gene expression in roots	5 weeks	[71]
shoots and roots	Co 51.91 mg kg^−1^, Cd 8.5 mg kg^−1^, Pb 436 mg kg^−1^	*Glomus mosseae* AMF	plant growth and nutrients take up	until early flowering	[74]
shoots and roots	Cd 0, 5, 10, mg kg^−1^	*Acrocalymma vagum* and *Scytalidium lignicola* DSE	plant growth, increase organic carbon level	60 and 90 days	[76]
leaves, roots	Pb 0, 60, 120, 180 and 240 μm	*Glomus intraradices* AMF	plant growth, protein, carotenoid, pigments, proline and total phenol content, enzyme activities	75 days	[77]
shoots and roots	Cd 20 mg kg^−1^	four AMF species and biochar	growth, nutrient and cadmium uptake	146 days	[79]
shoots and roots	Cd 300 mg kg^−1^ 600 mg kg^−1^ Zn 300 mg kg^−1^ 600 mg kg^−1^	*Rhizophagus irregularis* AMF and compost	plant growth, Photosynthetic efficiency, water content and membrane permeability	two months	[80]
shoots and roots	Cu 2.3 and 1.5 mM Pb 0.35 and 0.18mM Zn 4,3 and 2.15 mM	*Proteus* sp. DSP1, *Pseudomonas* sp. DSP17, *Ensifer meliloti* RhOL6 and RhOL8	plant growth, physiological state of the plants, nutrient composition of plants	two months	[80]
leaves, shoots and roots	Cr(VI) 100, 150, and 200 mg L^−1^	Four PGP and Cr (IV) resistance bacterial isolates	chlorophyll, proline, hydrogen peroxide, malondialdehyde content	5 weeks	[63]

## Data Availability

Data available on request.
